# An unusual presentation of spinal dural arteriovenous fistula: A case report

**Published:** 2016

**Authors:** Payam Saadat, Marzie Adabi

**Affiliations:** 1 Department of Neurology, Ayatollah Rouhani Hospital, Babol University of Medical Sciences, Babol, Iran.; 2 Department of Internal Medicine, Ayatollah Rouhani Hospital, Babol University of Medical Sciences, Babol, Iran

**Keywords:** Arteriovenous fistula, incontinence, Myelopathy

## Abstract

**Background:**

Spinal dural AVF is the most common type of spinal vascular malformation. However, presenting symptoms differ according to site of spinal involvement. This study described a case of arteriovenous malformation with paraparesis and incontinence.

**Case Presentation:**

Diagnosis of patient was confirmed by clinical and imaging examination using magnetic resonance image and ruling out other possibilities

**Result:**

A definitive diagnosis of arterio venous fistula was confirmed by clinical and MRI examination and demonstrated abnormalities compatible with dural arteriovenous fistula.

**Conclusion:**

Dural arteriovenous fistula should be considered in patients with paresis in both lower extremities.

Several pathological conditions such as degenerative, malignant, infectious and cystic or neuroligical causes may involve spine and results in spinal pain (1). In patients with suspected neurological diseases, detection of lesions require clinical and examination with emphasis on history. However, using magnetic resonance image is very helpful for diagnosis especialy in cases with spinal dural arteriovenous fistula (SDAVF) (2). Spinal malformations are frequent and were classified according to types of soft tissue injuries and spinal dural arteriovennous malformations who presented with limb paresis

## Case presentation

**Figure 1:**
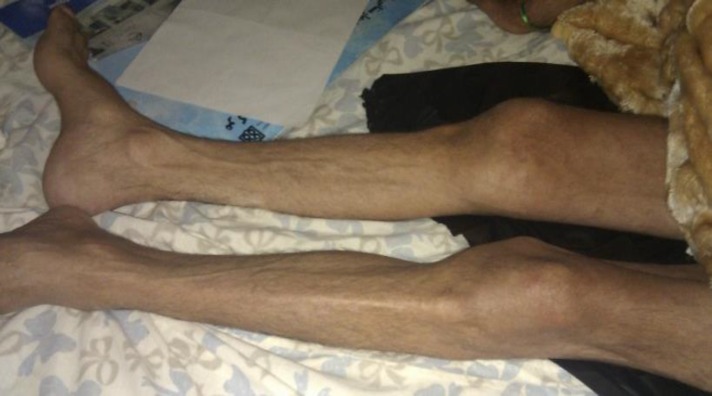
Bilateral atrophic lower extremities

A 51-year-old man presented with a 1-year history of right leg paresis with progressive numbness. Seven-months before this complaint, numbness of left lower extremity persisted. The patient began to experience occasional nocturnal urinary incontinence, fecal incontinency and stiffness of gait. He had a history of intermittent claudication during the last 3-years but had no history of radicular pain, low back pain or trauma. In neurological examination, the patient was oriented and cooperative. The cranial and upper extremities nerves were normal. The patients was unable to stand or walk and became bedridden. Examination of spine was normal without any limitations in movement or local tenderness. Muscle atrophy was seen in both of his lower extremities (figure 1) and muscle strength decreased significantly, whereas deep tendon reflexes were exaggerated in left lower extremity and hyporeflexic in right lower extremity. The flexor plantar reflex was in right foot but extensor in the left foot.

**Figure 2:**
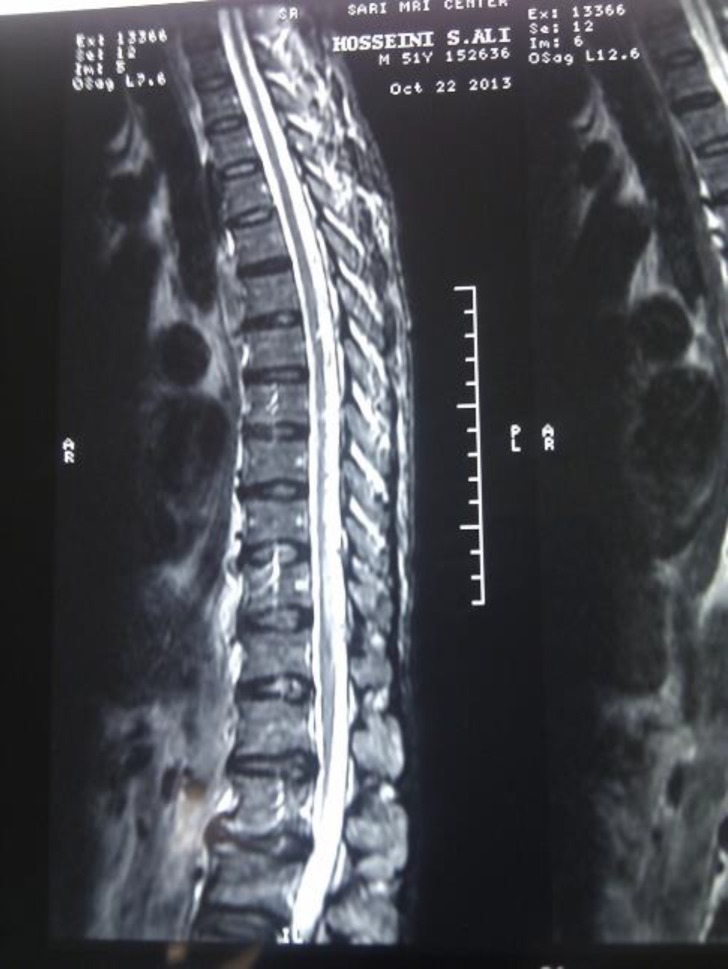
Sagittal T_2_- weighted magnetic resonance image of the thoracolumbar spine showing edema of the thoracic cord and regional dilated perimedullary vessels suggestive of a spinal vascular malformation

The patient's pinprick, light touch, vibration and position senses were impaired in both lower extremities. The results of magnetic resonance image (MRI) examination revealed regional dilated perimedulary vessels suggestive of dural AV fistula (figure 2). 

## Discussion

This patient presented with a history of progressive weakness on his both lower extrimities and signs and symptoms of myelopathy. The differential diagnosis for myelopathy is extensive; however, the main aim should be differentiated between structural and nonstructural lesions. Spinal dural ateriovenous fistulas are 9 times more common among men compared with women, with a mean age at diagnosis of 30-70 years, and the most frequent site of involvement is thoracolumbar region. This condition arises from an acquired abnormal communication of a branch of a segmental spinal artery with a radicular vein which is communicated with the perimedullary venous plexus of the spinal cord. High venous pressure in the arterialized vessels is thought to produce edema and relative ischemia of the spinal cord, which results in myelopathic symptoms.

Delayed diagnosis is common, and the time from disease onset to diagnosis ranges from 12 to 36 months (2). Progressive weakness, muscle spasm, fecal incontinence, overflow urinary incontinence or urinary retention and erectile dysfunction are characteristic of myelopathy, but they are not specific to spinal dural ateriovenous fistula. These symptoms may be aggravated by activities that increase intra-abdominal pressure. In about one-third of cases, lower motor neuron disease precedes upper motor neuron involvement (2). Sensory findings may follow an ascending radicular pattern.

Spinal dural ateriovenous fistula has a variable course ranging from acute onset that mimics anterior spinal artery syndrome to chronic and progressive symptoms (2). Untreated cases may progress to subacute necrosis of the spinal cord with permanent and severe impairment, including limb paralysis and loss of sphincter function (Foix–Alajouanine syndrome).

Treatment options include open spinal surgery to disconnect the arterialized draining vein or endovascular embolization; however, surgery has a higher likelihood of cure. In contrast, the first choice treatment for other spinal arteriovenous malformations is to use embolization (3). The outcome of the treatment depends on the age of the patient and the severity of preoperative myelopathy (4). Most patients show improvement in their symptoms and a reversal of radiologic changes after treatment (5). Gait difficulty and muscle strength respond better whereas, micturition, pain and muscle spasms respond less well (2).

A retrospective review by Gemmete et al, showed that lower extremity weakness was the most presenting feature of the disease. (7-9). Development of upper motor neuron signs with gait and micturation impairment occur at the later stage of the disease process. Similar to our reported case, involvement of thoracic spine has been reported too (8-10). In conclusion, these findings indicate that in patients with lower extremity paresis involvement of dural sac with subsequent neurological symptoms should be suspected and the site of abnormalities can be detected by appropriate clinical examination and imaging. 

## Citation:

Saadat P, Adabi M. An unusual presentation of spinal dural arteriovenous fistula: A case report. Caspian J Intern Med 2016; 7(3): 225-227.
